# Association of Regular Leisure-Time Physical Activity with Happiness among Middle-Aged and Older Adults in Taiwan

**DOI:** 10.3390/ijerph18158175

**Published:** 2021-08-02

**Authors:** Chyi Liang, Pei-Ling Wu, Po-Fu Lee, Chien-Chang Ho

**Affiliations:** 1 Department of Economics, Shih Hsin University, Taipei City 116, Taiwan; elsie219899@gmail.com; 2 Institute of Knowledge Economy Development, Shih Hsin University, Taipei City 116, Taiwan; peilin@mail.shu.edu.tw; 3 Department of Leisure Industry and Health Promotion, National Ilan University, Ilan County 260, Taiwan; f520184fred@yahoo.com.tw; 4 Department of Physical Education, Fu Jen Catholic University, New Taipei City 242, Taiwan; 5 Research and Development Center for Physical Education, Health, and Information Technology, Fu Jen Catholic University, New Taipei City 242, Taiwan; 6 Office of Physical Education, Fu Jen Catholic University, New Taipei City 242, Taiwan

**Keywords:** physical activity, exercise, mental health, happiness, middle-aged and older people

## Abstract

The aim of the present study was to clarify the relationship between regular LTPA (i.e., 150–300 min of moderate-intensity or 75–150 min of high-intensity physical activity) and happiness among middle-aged and older adults in Taiwan. The cross-sectional study data were obtained from the Taiwan National Physical Activity Survey, a nationally representative survey of the Taiwanese population. A total of 12,687 middle-aged and older adults (45–108 years) were ultimately enrolled in this study. The questionnaire data obtained through this national telephone survey included sociodemographic characteristics, self-reported health status, self-evaluations (comprising height, body weight, and happiness), and zip code of residence. The results suggest a significant positive relationship between regular LTPA and happiness scores; that is, the middle-aged adults who engaged in more LTPA may report higher happiness occurrence than others. This study suggests that regular LTPA is an essential factor influencing happiness. LTPA is an essential form of physical activity that helps middle-aged and older people to relax.

## 1. Introduction

Happiness is a subjective mental state characterized by feelings of enjoyment and satisfaction, and it reflects the overall subjective well-being of an individual [1]. In several countries such as Canada, France, and the United Kingdom, improvements in people’s overall happiness level are regarded as a sign of national progress [2,3]. Happiness also has positive effects on the long-term quality of relationships [4,5] and social interactions [6]. Moreover, in 32 countries, higher levels of life satisfaction and happiness were found to be related to lower suicide rates [7]. Higher levels of happiness are also related to lower mortality and morbidity [8,9]. The aforementioned findings constitute a part of a growing body of evidence supporting the essential role of happiness in people’s health.

Leisure-time physical activity (LTPA) has beneficial effects on physical and psychological health [10,11,12]. LTPA has also been established to have positive effects on quality of life [3,13]. In addition, studies have revealed the psychological benefits of LTPA in alleviating feelings of depression and anxiety [14,15]. LTPA not only promotes life satisfaction and happiness among adults aged 18–30 years but also has a positive relationship with perceived health [16] in healthy adults [17] (Downward and Dawson, 2016) and older adults [18]. Moreover, adults who engage in more LTPA enjoy higher levels of life satisfaction, and this effect is more pronounced in older adults relative to younger adults [19].

Considering the aforementioned findings, people may be inclined to consider engaging in regular LTPA. However, Taiwan’s Sports Administration [20] reported that 82.8% of the population participated in physical activity but only 33.0% regularly exercised. Studies exploring the relationship between age and happiness have produced inconsistent results; specifically, one study revealed an inverted U relationship [21] and another study discovered a U-shaped relationship, with lower physical activity levels from ages 45 to 54 but higher from ages 55 to 64 [22]. Such controversial arguments provide uncertain theories that warrant further examination. Furthermore, the association between regular LTPA and happiness has been less discussed previously. Therefore, the aim of the present study was to clarify the relationship between regular LTPA (i.e., 150–300 min of moderate-intensity or 75–150 min of high-intensity physical activity) and happiness among middle-aged and older adults in Taiwan.

## 2. Materials and Methods

### 2.1. Study Sample and Data Collection Procedures

The study data were obtained from the Taiwan National Physical Activity Survey (TNPAS), a nationally representative survey of children and adolescents (13–17 years), adults (18–64 years), and older adults (65 years and older) conducted by the Sports Administration, Ministry of Education, Taiwan. Enrollment was conducted through random-digit-dialing of individuals in a sample that was proportionally stratified by age, gender, and geographical district; the enrollment protocol was implemented in accordance with that used in another study [23]. The sample population consisted of citizens aged over 13 years and was stratified by city/county (22 cities/counties in Taiwan). The sample size for each city/county was calculated to reflect that city/county’s population as a proportion of Taiwan’s population. The initial total sample size for the present study was 25,526, with a sampling error range of 3–5% and confidence level of 95%; hence, a sufficient sample size and statistical power were achieved. Subsequently, a computer-assisted telephone interview (CATI) was conducted from August to October 2020 by a group of well-trained and experienced interviewers, who served to ensure the quality of data collection. The questionnaire data obtained through this national telephone survey comprised sociodemographic characteristics (age, gender, education, monthly income, and occupation), physical activity behavior, self-reported health status, self-evaluations (comprising height, body weight, and happiness), and zip code of residence. A total of 12,687 middle-aged and older adults (45–108 years) were ultimately enrolled in this study. The participants were fully informed about the objective, procedures, and contents of the present study, which was conducted in accordance with the Declaration of Helsinki and approved by the Institutional Review Board of Fu Jen Catholic University in Taiwan (FJU-IRB C109085). The participants provided oral consent prior to being interviewed. All relevant data are in a deidentified secondary dataset that has been made publicly available for research purposes.

### 2.2. Variables

The following demographic variables were recorded: age (45–49, 50–54, 55–59, 60–64, and ≥65 years), gender, education (elementary school or lower, junior or senior high school, and college or higher), monthly income, occupation (white collar, government servant, blue collar, owner/manager, specialist, student, housewife, retiree, freelancer, unemployed, and other), and self-reported health status (excellent or good, fair, very bad, and very poor). Anthropometric variables were obtained using the participants’ self-reported body height and weight data, and their body mass index (BMI, kg/m^2^) was calculated using these data. The cutoff BMI points for obesity were defined as: underweight, BMI < 18.5 kg/m^2^; normal weight, 18.5 ≤ BMI < 24 kg/m^2^; overweight, 24 ≤ BMI < 27 kg/m^2^; and obesity, BMI ≥ 27 kg/m^2^) [24].

### 2.3. LTPA

In the present study, regular or nonregular LTPA was determined through a series of questions during the CATI. First, the participants were asked to describe their current LTPA level. The question was as follows: “Have you participated in any LTPA in the past month?” Participants who provided a positive response were asked to describe their LTPA participation frequency and duration; the questions used were as follows: “How many times do you engage in LTPA every week?” and “How many minutes do you usually spend on each LTPA session?” Furthermore, LTPA intensity was assessed by asking the participants to describe their breathing and sweating when they were engaging in LTPA; the question asked was as follows: “When you are engaged in LTPA, you usually feel …”. In response, the participants structural answers such as “no changes in my breath or sweating”, “I breathe faster but do not sweat”, “I breathe normally but I sweat”, and “I breathe quickly and sweat”. Participants who reported that they usually breathed quickly and sweated were considered to have engaged in moderate-intensity LTPA. Subsequently, the participants were segmented into two groups. First, participants who participated in 150–300 min of moderate-intensity LTPA per week or 75–150 min of high-intensity LTPA per week and reported breathing quickly and sweating during LTPA were assigned to the regular LTPA group. The remaining participants were assigned to the nonregular LTPA group.

### 2.4. Happiness

The concepts and determinants of happiness are diverse [1,25,26,27]. Several methods are used to measure happiness, including the Oxford Happiness Inventory [28] and Satisfaction with Life Scale [29]. Nevertheless, these methods usually use a single-item question (i.e., “Taking all things together (in general), how happy would you say you are?”) to measure happiness in the LTPA domain [21,30,31,32,33,34]. This item has been validated [35,36] and tested for its temporal stability (test–retest reliability; r = 0.86) [37]. In TNPAS 2020, happiness was measured using the aforementioned single-item question and scored on a 5-point scale from 1 (“very unhappy”) to 5 (“very happy”). Furthermore, to establish binary data, the respondents were dichotomized into happy (i.e., very happy, happy, and fair) and unhappy (i.e., unhappy and very unhappy) groups according to the protocol used in another study [38].

### 2.5. Statistical Analysis

IBM SPSS 25.0 (IBM Co., Armonk, NY, USA) was used for this study. Student’s *t*-test was performed to compare the continuous variables of the regular and nonregular LTPA groups. A Chi-square test was used to examine the associations between the regular and nonregular LTPA groups and the associations among their categorical variables. Multiple linear regression, with the happiness score being the dependent variable, was performed to examine the association between regular LTPA and happiness scores after adjustment for potential confounders. Unconditional logistic regression models were applied to calculate the adjusted odds ratios for happiness. Analysis results are presented herein as mean ± standard deviation or as numbers (frequency percentages). A result was considered statistically significant at a two-tailed *p*-value of < 0.05.

## 3. Results

Table 1 shows the research population’s demographic characteristics. A total of 12,687 participants were categorized into two groups according to their engagement in LTPA. Of the participants, >20% were assigned to the regular LTPA group. Compared with the nonregular LTPA group, the regular LTPA group had a higher proportion of men (53.3%), higher percentage of participants in the normal weight range (48.8%), and higher level of education (27% received at least college education); approximately 34.9% of the participants in this group were already retired, and 75.5% self-reported that they were in excellent or good health. The two groups differed significantly in all demographic data, except for BMI.

Table 2 presents a comparison of the happiness scores of the participants in the regular and nonregular LTPA groups. Both men and women in the regular LTPA group reported higher happiness scores than did those in the nonregular LTPA group; moreover, a significant difference was observed between these two groups with respect to the happiness scores reported by the 50–54 age group (*p* < 0.05). Among men in the 60–64 and ≥65 age groups, a significant difference in happiness scores was observed between the regular and nonregular LTPA groups (*p* < 0.05). Among women in the 45–49 and 55–59 age groups, a significant difference in happiness scores was observed between the regular and nonregular LTPA groups. Significant differences were observed (*p* < 0.05) for all comparisons that used pooled data.

Table 3 shows a comparison of the prevalence of happiness/unhappiness between the regular and nonregular LTPA groups. In both groups, significant differences in the prevalence of happiness and unhappiness were observed among the age groups (*p* < 0.05). Among women in the 45–49 and 50–54 age groups, significant differences in the prevalence of happiness and unhappiness were observed between the regular and nonregular LTPA groups. Among men in the 50–54, 60–64, and ≥65 age groups, significant differences in the prevalence of happiness and unhappiness were observed between the regular and nonregular LTPA groups. Furthermore, both the regular and nonregular LTPA groups exhibited a higher prevalence of unhappiness relative to happiness.

Table 4 presents the multivariate linear regression results for the happiness scores of the regular LTPA group. The results indicated a significant positive relationship between engagement in regular LTPA and happiness (*p* < 0.05). After adjustments for age, education, occupation, obesity status, and self-reported health status, the explanatory power of the significantly decreased for both men and women (β = 0.028 for men, β = 0.045 for women).

Table 5 provides the results from the multiple logistic regression models. The LTPA population was planned as the reference for all analyses (OR = 1.000). The results from model 2 indicated that male and female participants engaged in regular LTPA had better likelihood to report they were happy (OR = 1.216 for men, OR = 1.401 for women; *p* < 0.05).

## 4. Discussion

This study explored the association of regular LTPA with happiness among 12,687 middle-aged and older adults in Taiwan. The results suggest a positive relationship between regular LTPA and happiness; that is, the middle-aged adults who engage in more LTPA may have higher happiness occurrence than others. In particular, in the regular LTPA group, both men and women reported relatively high happiness. In both the regular and nonregular LTPA groups, the prevalence of happiness and unhappiness was significantly different among age groups. However, in both the regular and nonregular LTPA groups, the prevalence of happiness and unhappiness was significantly different in two female age groups (45–49 and 50–54) and three male age groups (50–54, 60–64, and ≥65).

These findings provide evidence of how multiple factors influence the relationship between regular LTPA and happiness among middle-aged and older people. Previous studies have produced similar results regarding the association of LTPA with life satisfaction [16,19] and quality of life [39,40]. Hence, further research on the direct and indirect influence of LTPA on happiness may help clarify the discrepancies pertaining to age- and sex-related trends. Some studies have identified age as a factor that reduces LTPA [41,42], but others have suggested age to be associated with an increasing or stable trend [43] or diversification patterns of LTPA [44,45,46]. Therefore, the existence of other moderating or mediating variables cannot be ruled out.

Furthermore, in both the regular and nonregular LTPA groups, the prevalence of happiness and unhappiness did not differ significantly between the two genders. A previous study discovered gender to be associated with longer television viewing time and poorer cardiovascular health, which was observed even among participants who met physical activity recommendations; this observation could not be explained [47]. Future studies may reveal previously unknown underlying mechanisms or verify the absence of gender differences.

The strength of this study was using a huge sample size to analyze the relationship between regular LTPA and happiness among Taiwanese middle-aged adults and elders. However, there are some limitations that should be carefully addressed. First, the use of a secondary database meant that other potential confounders such as chronic diseases or mental disorders could not be considered. Furthermore, the daily activity levels were not able to take part in our analysis. Specifically, the level of activity of daily living (ADL) might be crucially confounding one’s quality of late-life, as well as his/her LTPA participation. In other words, the gap may have existed in the LTPA–happiness relationship, particularly for the individuals who have a low ADL level. Therefore, future research should conduct comprehensive investigations that allow analyses of any possible confounders. Second, this study did not examine low-intensity LTPA and might have overlooked the differences engendered by LTPA of varying intensities. Third, the study used a cross-sectional design and was thus unable to verify the existence of a causative relationship. Fourth, although a huge data set was used, the data collected from the CATI was based on self-reported information. The trustworthiness of the data may not be guaranteed.

## 5. Conclusions

In summary, this study revealed that regular LTPA is an essential factor influencing happiness. LTPA is an essential form of physical activity that helps people to relax. Therefore, regular LTPA is crucial for middle-aged and older people, who should be encouraged to increase the duration and intensity of their LTPA.

## Figures and Tables

**Table 1:** Demographic characteristics.

Variables	LTPA Status		*p*-Value
	Regular LTPA (*n* = 2641)	Nonregular LTPA (*n* = 10,046)	
Age (y) ^b^			0.037 *
45–49	427 (16.20%)	1655 (16.50%)	
50–54	480 (18.20%)	1647 (16.40%)	
55–59	434 (16.40%)	1721 (17.10%)	
60–64	438 (16.60%)	1525 (15.20%)	
≥65	862 (32.60%)	3497 (34.80%)	
Gender (% men) ^b^	1407 (53.3%)	4452 (44.3%)	<0.0001 *
Height (cm) ^a^	163.62 ± 7.78	161.92 ± 7.95	<0.0001 *
Body weight (kg) ^a^	64.60 ± 11.61	62.88 ± 11.63	<0.0001 *
BMI (kg/m^2^) ^a^	24.04 ± 3.38	23.90 ± 3.46	0.063
Obese Status (%) ^b^			<0.0001 *
Underweight	53 (2.00%)	310 (3.10%)	
Normal weight	1289 (48.80%)	4805 (47.80%)	
Overweight	770 (29.20%)	2668 (26.60%)	
Obese	407 (15.40%)	1572 (15.60%)	
Education (%) ^b^			<0.0001 *
Elementary school and lower	340 (13.00%)	1904 (19.10%)	
Junior or senior high	1570 (60.00%)	6100 (61.30%)	
College and higher	706 (27.00%)	1940 (19.50%)	
Occupation (%) ^b^			<0.0001 *
White collar	287 (10.90%)	894 (9.00%)	
Government servant	161 (6.10%)	408 (4.10%)	
Blue collar	323 (12.30%)	1931 (19.40%)	
Owner/manager	194 (7.40%)	630 (6.30%)	
Specialists	104 (4.00%)	353 (3.50%)	
Student	0 (0.00%)	2 (0.00%)	
Housewife	433 (16.50%)	2124 (21.40%)	
Retired	917 (34.90%)	2790 (28.00%)	
Free lancer	121 (4.60%)	445 (4.50%)	
Jobless	69 (2.60%)	328 (3.30%)	
Other	15 (0.60%)	42 (0.40%)	
Self-reported health status (%) ^b^			<0.0001 *
Excellent or good	1967 (77.50%)	6826 (71.90%)	
Fair	184 (7.30%)	902 (9.50%)	
Very bad or poor	386 (15.20%)	1767 (18.60%)	

Abbreviations: BMI, body mass index; LTPA, leisure-time physical activity. * *p* < 0.05. ^a^ Values expressed as mean ± standard deviation. ^b^ Values expressed as n (percentage).

**Table 2:** Comparison of happiness scores between regular and nonregular LTPA groups among middle-aged and older adults in Taiwan.

Variables	LTPA Status		*p*-Value
	Regular LTPA	Nonregular LTPA	
**Men (*n* = 5557) ^a^**			
45–49	3.78 ± 0.78	3.68 ± 0.80	0.104
50–54	3.85 ± 0.55	3.67 ± 0.78	<0.0001 *
55–59	3.82 ± 0.69	3.75 ± 0.78	0.163
60–64	3.98 ± 0.59	3.83 ± 0.74	0.002 *
≥65	3.95 ± 0.65	3.86 ± 0.69	0.013 *
**Women (*n* = 6540) ^a^**			
45–49	3.89 ± 0.61	3.75 ± 0.73	0.002 *
50–54	4.02 ± 0.60	3.77 ± 0.73	<0.0001 *
55–59	3.92 ± 0.67	3.81 ± 0.74	0.038 *
60–64	3.94 ± 0.63	3.85 ± 0.68	0.088
≥65	3.95 ± 0.63	3.90 ± 0.69	0.232
**Pooled (*n* = 12,097) ^a^**			
45–49	3.84 ± 0.69	3.72 ± 0.76	0.001 *
50–54	3.93 ± 0.58	3.73 ± 0.75	<0.0001 *
55–59	3.87 ± 0.68	3.79 ± 0.76	0.021 *
60–64	3.96 ± 0.61	3.84 ± 0.71	0.001 *
≥65	3.95 ± 0.64	3.88 ± 0.69	0.011 *

Abbreviations: LTPA, leisure-time physical activity. * *p* < 0.05. ^a^ Values expressed as mean ± standard deviation.

**Table 3:** Comparison of happiness/unhappiness prevalence between regular and nonregular LTPA groups among middle-aged and older adults in Taiwan.

Variables		LTPA Status		*p*-Value
		Regular LTPA	Nonregular LTPA	
**Men (*n* = 5558) ^a^**				
45–49	Happy	31 (15.50%)	150 (20.90%)	0.089
	Unhappy	169 (84.50%)	567 (79.10%)	
50–54	Happy	34 (13.50%)	161 (25.00%)	<0.0001 *
	Unhappy	217 (86.50%)	482 (75.00%)	
55–59	Happy	36 (16.60%)	152 (21.40%)	0.124
	Unhappy	181 (83.40%)	559 (78.60%)	
60–64	Happy	22 (9.80%)	113 (17.50%)	0.006 *
	Unhappy	202 (90.20%)	534 (82.50%)	
≥65	Happy	64 (13.60%)	260 (17.60%)	0.038 *
	Unhappy	408 (86.40%)	1214 (82.40%)	
**Women (*n* = 6539) ^a^**				
45–49	Happy	26 (11.60%)	188 (20.60%)	0.002 *
	Unhappy	199 (88.40%)	726 (79.40%)	
50–54	Happy	18 (8.10%)	187 (19.60%)	<0.0001 *
	Unhappy	205 (91.90%)	767 (80.40%)	
55–59	Happy	28 (13.30%)	176 (19.00%)	0.055
	Unhappy	182 (86.70%)	752 (81.00%)	
60–64	Happy	25 (12.10%)	127 (16.00%)	0.169
	Unhappy	181 (87.90%)	667 (84.00%)	
≥65	Happy	45 (13.30%)	257 (14.70%)	0.492
	Unhappy	294 (86.70%)	1490 (85.30%)	
**Pooled (*n* = 12,097) ^a^**				
45–49	Happy	57 (13.40%)	338 (20.70%)	0.001 *
	Unhappy	369 (86.60%)	1292 (79.30%)	
50–54	Happy	52 (11.00%)	349 (21.80%)	<0.0001 *
	Unhappy	422 (89.00%)	1249 (78.20%)	
55–59	Happy	63 (14.80%)	328 (20.00%)	0.013 *
	Unhappy	364 (85.20%)	1311 (80.00%)	
60–64	Happy	47 (10.90%)	239 (16.60%)	0.004 *
	Unhappy	383 (89.10%)	1202 (83.40%)	
≥65	Happy	109 (13.40%)	517 (16.10%)	0.066
	Unhappy	702 (86.60%)	2703 (83.90%)	

Abbreviations: LTPA, leisure-time physical activity. * *p* < 0.05. ^a^ Values expressed as *n* (percentage).

**Table 4:** Multivariate regression of happiness scores of regular LTPA group.

Variables	Model 1 (Unadjusted)			Model 2 (Adjusted ^a^)		
	*β*	SE	*p*-Value	*β*	SE	*p*-Value
**Men**						
Regular LTPA	0.059	0.020	<0.0001 *	0.028 ^a^	0.020	0.039 *
Nonregular LTPA	Ref.	-	-	Ref.	-	-
**Women**						
Regular LTPA	0.071	0.024	<0.0001 *	0.045 ^a^	0.023	0.001 *
Nonregular LTPA	Ref.	-	-	Ref.	-	-
**Total**						
Regular LTPA	0.061	0.015	<0.0001 *	0.030 ^b^	0.015	0.002 *
Nonregular LTPA	Ref.	-	-	Ref.	-	-

Abbreviations: LTPA, leisure-time physical activity; SE, standard error. * *p* < 0.05. ^a^ Adjusted for age, education, occupation, obesity status, and self-reported health status. ^b^ Adjusted for age, gender, education, occupation, obesity status, and self-reported health status.

**Table 5:** Multivariate logistic regression of happiness status of regular LTPA group.

Variables	Model 1 (Unadjusted)			Model 2 (Adjusted)		
	OR	95% CI	*p*-Value	OR	95% CI	*p*-Value
**Men**						
Regular LTPA	1.470	1.239–1.743	<0.0001 *	1.216 ^a^	1.017–1.453	0.032 *
Nonregular LTPA	Ref.	-	-	Ref.	-	-
**Women**						
Regular LTPA	1.677	1.314–2.141	<0.0001 *	1.401 ^a^	1.089–1.804	0.009 *
Nonregular LTPA	Ref.	-	-	Ref.	-	-
**Total**						
Regular LTPA	1.493	1.300–1.714	<0.0001 *	1.221 ^b^	1.057–1.409	0.007 *
Nonregular LTPA	Ref.	-	-	Ref.	-	-

Abbreviations: CI, confidence interval; LTPA, leisure-time physical activity; OR, odds ratio. * *p* < 0.05. ^a^ Adjusted for age, education, occupation, obesity status, and self-reported health status. ^b^ Adjusted for age, gender, education, occupation, obesity status, and self-reported health status.

## Data Availability

The design and data used in this study were approved and provided by the Sports Cloud: Information and Application Research Center of Sports for All, Sport Administration, Ministry of Education, Taiwan.
